# The Short-Term Efficacy of Large-Focused and Controlled-Unfocused (Radial) Extracorporeal Shock Wave Therapies in the Treatment of Hip Osteoarthritis

**DOI:** 10.3390/jpm13010048

**Published:** 2022-12-26

**Authors:** Volkan Şah

**Affiliations:** Department of Sports Medicine, University of Yüzüncü Yıl, Van 65040, Turkey; volkansah@yyu.edu.tr

**Keywords:** hip osteoarthritis, ESWT, radial, focused

## Abstract

Although the classical treatments listed in the guidelines for osteoarthritis are widely used, the majority of patients do not fully recover from their pain. It is a fact that new treatment methods are needed both to relieve pain and restore deteriorated joint function. No study has been found to date that evaluated the efficacy of ESWT in hip OA. This pilot trial is the first in the literature to investigate the comparative effects of the two ESWT types (f-ESWT and r-ESWT) in the treatment of hip OA. Briefly, 148 patients were randomly distributed into the three ESWT groups: focused (f-ESWT), radial (r-ESWT), and sham (s-ESWT). Patients were assessed with the Visual Analog Scale (VAS) and Western Ontario and McMaster Universities Osteoarthritis Index (WOMAC) scores just before the treatment (0 week), just after the treatment (4th week), and 1 month after completion of the treatment (8th week). VAS and all WOMAC scores were significantly reduced at follow-up points (4th and 8th weeks) in both the f-ESWT and r-ESWT groups compared with baseline (0 week) (for all, *p* < 0.001). Statistical comparisons between the f-ESWT and r-ESWT groups showed that f-ESWT was superior to r-ESWT for the decrease in VAS and WOMAC scores from baseline to the 4th and 8th weeks (*p* < 0.001 or *p* < 0.002). Both r-ESWT and f-ESWT were found to have significant treatment efficacy compared with s-ESWT. However, f-ESWT produced a superior improvement in follow-up parameters compared to r-ESWT.

## 1. Introduction

Osteoarthritis (OA) is a progressive joint disease that starts with disruptions in joint tissue metabolism, continues with cartilage destruction, sclerosis formation under the cartilage, osteophyte formation, and inflammation in the joint and eventually impairs joint function [1]. Older age and female gender have been shown to be risk factors in hip osteoarthritis (OA) [2]. While 28% of people over the age of 45 have radiological signs of hip OA, 9.7% of these people complain of HOA symptoms [3]. A history of previous trauma (especially hip, knee, and ankle), and congenital or acquired anatomical deformities are other risk factors for hip OA [3,4].

Exercise, patient education, and physical therapy modalities are used as non-pharmacological treatments for hip OA. Paracetamol, NSAIDs, and intra-articular steroid injections are pharmacological options [5]. In cases unresponsive to these medical treatments, total hip replacement is applied as a surgical method [6]. Although classical treatments are widely used in the treatment guidelines for osteoarthritis [7], the majority of patients do not fully recover from their pain [8]. It is a fact that new treatment methods are needed both to relieve pain and restore deteriorated joint function.

Extracorporeal shock wave therapy (ESWT), which had started to be used for the treatment of musculoskeletal disorders [9,10,11,12], was also found to be efficient in OA as a non-invasive, non-pharmacological treatment method with a low complications rate in a study conducted by Kim et al. [13]. In ESWT, high-intensity pressure waves are applied to the body surface. As shock waves pass through different tissues, some of their energy is transmitted to the tissue and some is reflected. Micro-level changes are seen according to the physical properties of the tissue [11].

Two different types of shock waves are used for the treatment of musculoskeletal disorders: focused (f-ESWT) and radial (r-ESWT). While f-ESWT reaches the highest energy density in the depths of the tissue to which it is applied, the energy of r-ESWT decreases in proportion to the distance of the tissue from the surface [14].

The anti-inflammatory, anti-apoptotic, new-small-vessel-forming, and regenerative effects of ESWT, which have been shown in animal studies, suggest that it may be effective in the treatment of OA [15,16,17,18,19]. A novel meta-analysis of 14 studies with 782 participants demonstrated that ESWT is an effective treatment for improving functional status and pain in patients with knee OA. Of the 14 studies, 5 used f-ESWT, 8 used r-ESWT, and 1 used both, but none were “ESWT type comparison” studies [20]. In a very recent study comparing f-ESWT and r-ESWT in the treatment of knee OA, although both were observed to be effective, f-ESWT was superior to r-ESWT in reducing pain and improving function [21].

Despite the intense research about the treatment of knee OA with ESWT, hip OA has been overshadowed by this interest. Only a veterinary study showed that r-ESWT improved limb function in dogs with hip OA [22]. To the best of our knowledge, there has been no study to date that evaluates either the efficacy of ESWT or the comparison of focused and radial wave types in the treatment of human hip OA. This pilot study will try both to find out whether ESWT is effective in the treatment of patients with hip OA and to compare the efficacy of the two ESWT types (f-ESWT and r-ESWT).

## 2. Materials and Methods

### 2.1. Ethical Considerations

This study was carried out at Van Yüzüncü Yıl University, Department of Sports Medicine, Turkey. All patients were informed verbally before the study, and all of them filled out written informed consent forms in accordance with the Declaration of Helsinki. Yüzüncü Yıl University Clinical Research Ethics Committee approval (Decision No: 06; 2 March 2022) and ‘Clinicaltrials.gov’ registration (no: NCT05224674) were obtained.

### 2.2. Study Design

This was a prospective, double-blind, randomized, and sham-controlled clinical trial comparing the large-focused ESWT (f-ESWT) and controlled-unfocused/radial ESWT (r-ESWT) treatment groups. The study lasted for 8 weeks, of which the first 4 weeks were the treatment period. During the first 7 days of the study, all patients were given an oral analgesic (paracetamol 500 mg tablets) twice daily. Patients were assessed with Visual Analog Scale (VAS) and Western Ontario and McMaster Universities Osteoarthritis Index (WOMAC) scores just before the treatment (0 week), just after the treatment (4th week), and 1 month after the end of treatment (8th week). In patients with bilateral pain, treatments were applied to both sides, but evaluations were made based on the most painful side.

### 2.3. Estimation of Sample Size and Study Power

Study power and sample size values were calculated with the G*Power statistical program (version 3.1.9.7, Heinrich-Heine Universität Düsseldorf, Düsseldorf, Germany). The study power was calculated based on the sample size. For each group, a total of 51 patients were provided to achieve an effect size of 0.5, a power of 0.80, and a significance level of 0.05.

### 2.4. Blinding

The patients were not informed about the sequence of procedures and their differences from each other. An academician who was not involved in the study randomly assigned the participants to the treatment groups. The treatments were applied by the physical therapy technicians, who did not participate in patient distribution and were blinded to the treatment follow-up records. The patients did not realize which treatment group they were included in since ESWT had never been applied to them before, and similar pulse sounds were heard in all three groups of treatment. Researchers who did not participate in the collections evaluated the results. This allowed the outcome evaluation to be blinded, which reduced the possibility of the study’s detection bias. In addition, all results were fully recorded.

### 2.5. Participants

**Figure 1:**
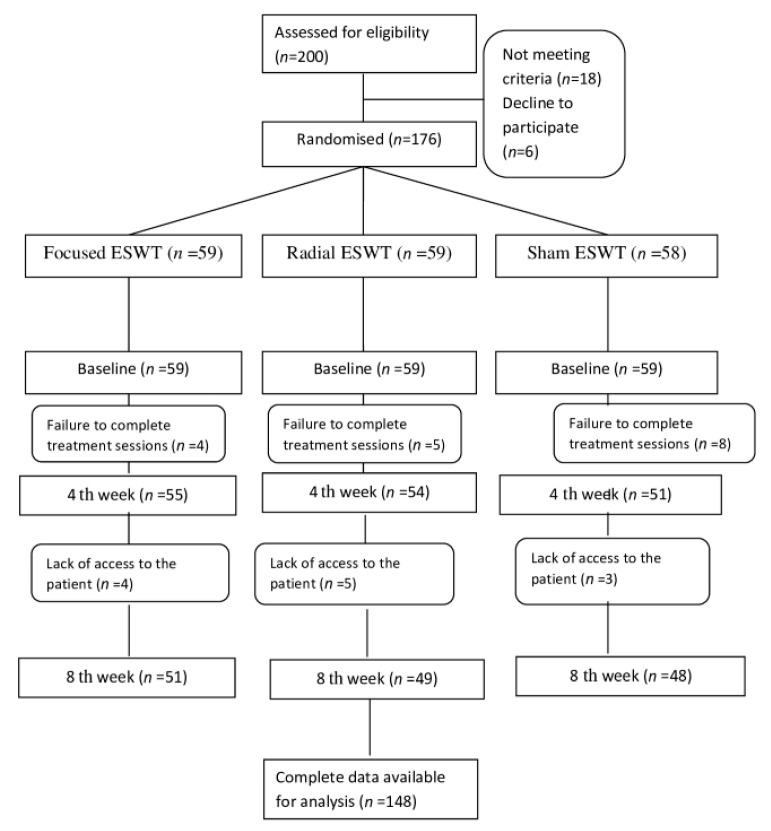
Flowchart of the study participants.

In total, 200 patients with unilateral or bilateral hip osteoarthritis evaluated in the Van Yüzüncü Yıl University Sports Medicine outpatient clinic (admitted directly or referred from other outpatient clinics) were evaluated in terms of inclusion and exclusion criteria. The statistical results of 148 patients who accepted to participate in the study, met the criteria, and were able to be evaluated at the last control were analyzed. Figure 1 shows the flowchart of the participants.

### 2.6. Inclusion/Exclusion Criteria

The inclusion criteria were being over 50 years old, having symptoms that have persisted for at least 6 months (unilateral or bilateral), and having fulfilled the hip OA diagnostic criteria according to clinical plus radiographic criteria—hip pain with at least 2 of the following 3 criteria—femoral or acetabular osteophyte, superior/axial/medial joint space narrowing, and erythrocyte sedimentation rate < 20 mm/h—set forth by the American College of Rheumatology [23], and having grade 2 and 3 changes in pelvic anterior-posterior graphy according to Kellgren–Lawrence classification [24].

The exclusion criteria were previous ESWT treatment, systemic comorbid diseases such as hypertension and diabetes, rheumatological and other mechanical diseases of the hips, coagulation diseases, malignancies, infections, body implants, pacemakers, pregnancy, previous surgical interventions of the hip or intra-articular injections in the last 6 months, and neurological and psychiatric diseases in which cooperation is impaired.

### 2.7. Randomization

In total, 148 patients were assigned to the groups by “block randomization” with the help of the “Random Allocation Software (ver.1.0)” package program.

### 2.8. Interventions

**Figure 2:**
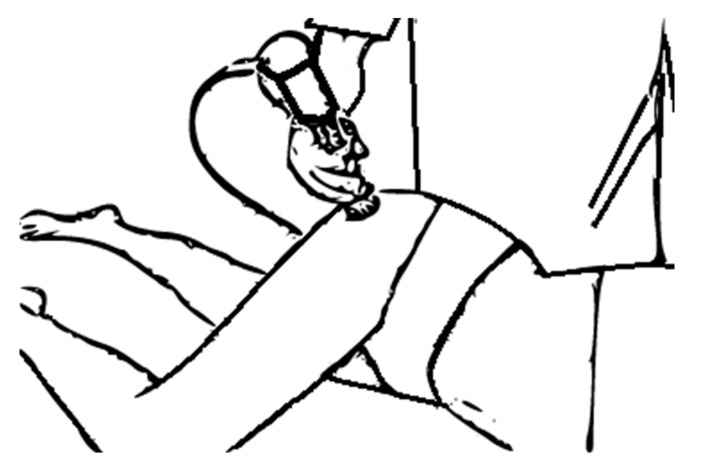
Schematic application of ESWT.

The ESWT application consisted of a total of four sessions administered at one-week intervals. In the lying lateral decubitus position, by bringing the hip and knee to flexion angle of 90 degrees, ESWT was applied topographically to the coxofemoral joint from the lateral to the medial with ultrasound gel without using local anesthesia. Figure 2 shows schematically the application of ESWT.

The same ESWT device was used in all sessions and in both treatment groups (Pagani Elettronica, made in Italy). The device works with an electro-pneumatic system and has the ability to generate both radial and focused waves. The frequency, pressure, energy, pulse, and duration values when hip osteoarthritis diagnosis is selected on the screen were as follows:

The f-ESWT was applied in 2 consecutive parts in each session; part 1 (4 Hz, 1.6 Bar, 500 pulses, 0.02–0.60 mJ/mm^2^, 2 min 5 s) + part 2 (8 Hz, 1.8 Bar, 1500 pulses, 0.02–0.60 mJ/mm^2^, 3 min 8 s).

The r-ESWT was applied in 2 consecutive parts in each session; part 1 (6 Hz, 1.5 Bar, 500 pulses, 0.180 mJ/mm^2^, 1 min 23 s) + part 2 (8 Hz, 1.6 Bar, 1500 pulses, 0.192 mJ/mm^2^, 3 min 8 s). 

The sham (s-ESWT) was applied with the r-ESWT probe. Even though the frequency (Hz), pressure (Bar), and duration (minute) values were the same as in r-ESWT, the energy level (joule) was manually set to 0 (zero) in order not to apply energy to the patient.

### 2.9. Outcome Measures

VAS was used to assess the pain intensity of the patients. This scale consists of a horizontal line with the value of 0 (zero) at the beginning of the line, and 10 (ten) at the end. The patient is asked to mark the intensity of pain at rest on this scale. A value of 0 is considered to mean no pain, and numbers that go up to 10 represent an increase in pain level, with a value of 10 being considered unbearable pain. This scale was first used in psychology by Freyd in 1923 [25].

WOMAC was used for the pain and functional assessments of the patients. It is a scale that evaluates the disability associated with hip and/or knee osteoarthritis. It was first described in 1984 to standardize the outcomes of osteoarthritis [26]. It consists of 3 parts: pain, stiffness, and physical function, with a total of 24 items. Items are scored on a Likert scale. The degree of pain and disability is indicated by giving points from 0 to 4 on the Likert scale. High Womac scores indicate an increase in pain and stiffness and a deterioration in physical function. The Turkish validity and reliability study was accomplished by Tüzün et al. [27]. 

### 2.10. Statistical Analysis

Statistical analyses were performed using the SPSS v20 program (IBM Corp., Armonk, NY, USA). Whether the variables were normally distributed or not was determined by the Kolmogorov–Smirnov test. The following scores, changing from baseline to follow-up periods, were found to be non-normally distributed:

- In the f-ESWT Group: VAS (baseline–4th week) and WOMAC stiffness (baseline–4th week);
- In the r-ESWT Group: VAS (baseline–8th week), WOMAC stiffness (baseline–4th week), and WOMAC stiffness (baseline–8th week);
- In the s-ESWT Group: VAS (baseline–4th week), VAS (baseline–8th week), WOMAC pain (baseline–4th week), WOMAC pain (baseline–8th week), WOMAC stiffness (baseline–8th week), WOMAC function (baseline–8th week), and WOMAC total (baseline–8th week).

Therefore, the non-parametric tests (Kruskal–Wallis and Mann–Whitney U) were used for these scores in the statistical comparisons. For the other scores with normal distributions, the parametric tests (repeated measures ANOVA, one-way ANOVA with Bonferroni, and independent samples *t* test) were used in the statistical comparisons. Therefore, the non-parametric tests (Kruskal–Wallis and Mann–Whitney U) were used for these non-normally distributed scores in the statistical comparisons. For the other scores with normal distribution the parametric tests (paired *t*, one-way ANOVA with Bonferroni, and independent samples *t*) were used in the statistical comparisons. The paired *t* test was used for intra-group comparisons between scores obtained at the baseline and at the follow-up periods. The Kruskal–Wallis and one-way ANOVA tests were used to compare the scores of three groups, and then the Bonferroni, Mann–Whitney U, and independent samples *t* tests were used to compare the scores of two groups.

Categorical variables, which have been represented as numbers, were analyzed using the chi-square test. Continuous variables were shown as mean ± standard deviation (min–max). Results with ‘*p* < 0.05’ were considered statistically significant.

## 3. Results

Table 1 presents the participants’ characteristics. The groups were similar with respect to baseline characteristics (age, gender, body mass index, radiological grade, and pain duration) (for all, *p* > 0.05) (Table 1). Additionally, the three groups were similar in terms of baseline VAS and WOMAC scores for pain, stiffness, function, and total points (for all, *p* > 0.05) (Table 2, Table 3, Table 4, Table 5 and Table 6).

The paired *t* tests showed that at the follow-up periods (weeks 4 and 8), all VAS were significantly reduced in both focused and radial ESWT groups compared with baseline (week 0) (for all, *p* < 0.001). Additionally, the paired *t* tests showed that all subscores and total scores of WOMAC were significantly reduced in both focused and radial ESWT groups compared with baseline (week 0) (for all, *p* < 0.001) (Table 2, Table 3, Table 4, Table 5 and Table 6).

However, the scores obtained at weeks 4 and 8 were similar to baseline in the sham ESWT group (for all, *p* > 0.05) (Table 2, Table 3, Table 4, Table 5 and Table 6), except the WOMAC pain score from baseline to week 8, which had even increased (*p* = 0.021) (Table 3), demonstrating pain aggravation in the sham ESWT group.

When considering the change of scores from baseline to the follow-up periods (weeks 4 and 8), statistical comparisons showed that both focused ESWT and radial ESWT groups were significantly superior to the sham ESWT group in the all of VAS and WOMAC scores (*p* < 0.001) (Table 2, Table 3, Table 4, Table 5 and Table 6), except the change of WOMAC function score from baseline to weeks 4 (*p* < 0.077) (Table 5), which shows radial ESWT and sham ESWT groups have similar effects at that point.

Importantly, statistical comparisons between the f-ESWT and r-ESWT groups showed that f-ESWT was superior to r-ESWT for change in VAS and WOMAC scores from baseline to the 4th and 8th weeks (*p* < 0.001 or *p* < 0.002) (Table 2, Table 3, Table 4, Table 5 and Table 6).

No side effects were observed in any of the participants as a result of the paracetamol or ESWT treatments.

## 4. Discussion

This randomized, controlled, double-blind study demonstrated that ESWT could be an effective and safe method to control pain and improve functional status in patients diagnosed with hip OA. To the best of our knowledge, this is the first study about the efficacy of treating human hip OA with ESWT. According to the pain and joint function assessment using VAS and WOMAC scores, both f-ESWT and r-ESWT produced significant improvements in the treatment of hip osteoarthritis. However, f-ESWT produced a superior improvement in these scores compared to R-ESWT.

Osteoarthritis (OA) is a chronic degenerative disease common enough to affect approximately one-third of adults aged 65 and over. Hip and knee OA are the most common types of OA. Physical therapy is widely used in the treatment of hip OA [5]. However, definite treatment protocols with clearly proven efficacy have not been established. Therefore, extracorporeal shock wave therapy (ESWT), which has a newer technology compared to other physical therapy modalities, seems to be a promising alternative [28].

ESWT is known to be effective in some musculoskeletal diseases and especially in the treatment of enthesopathies, including plantar fasciitis, elbow tendinitis, epicondylitis, patellar tendinitis, and Achilles tendinitis [11]. The precise mechanisms of ESWT’s pain-relieving effect are unknown; however, it has been proposed that ESWT improved tissue healing by increasing TGF-ß1and IGF-1 expression and that it may also have a neovascularization-inducing effect by increasing the release of vascular endothelial growth factor and endothelial nitric oxide synthase [29].

From the first article in 2013 [30] to date, previous studies on the treatment of OA with ESWT have mostly focused on knee OA [21,30,31,32,33,34,35,36]. 

In an RCTs where the 105 female patients were followed up to 3 months post-treatment, Imamura et al. compared r-ESWT with s-ESWT in the treatment of knee OA and found that there was no significant difference between these two treatments in the change of WOMAC scores and VAS values, except for the WOMAC pain sub-score. The energy level of the r-ESWT they used increased up to a maximum of 0.16 mj/mm^2^, and they stated that higher energy levels might be needed for more successful treatment results [32]. Similarly, in another study using low-energy ESWT, the 2.5 bar pressure used in the treatment ESWT group was reduced to 0.2 bar in the s-ESWT group. The treatment group showed significantly superior improvement in VAS and WOMAC scores and the Lequesne index compared to the s-ESWT group [34].

Uysal et al. applied r-ESWT with 2000 shocks, 10 Hz frequency, and 2–3 bar pressure and s-ESWT (*n* = 52 patients) with 0 shocks, 10 Hz frequency, and 0.1 bar pressure in RCTs VAS, WOMAC, Lequesne, 20 m walk test, and knee ROM scores up to 3 months after treatment, revealing that r-ESWT was significantly superior to s-ESWT [35]. In a study where the r-ESWT was applied in 89 patients in 4 different combinations according to energy levels (0.12 mj/mm^2^ and 0.24 mj/mm^2^) and shocks (2000 and 4000), and the same number of shocks in which the energy level was reduced to 0.02 mj/mm^2^ (sham group), treatment results were compared immediately after the treatment and 4 weeks after the treatment. It was revealed that there was no significant difference between 2000 shocks and 4000 shocks, but significantly more effective treatment results were obtained in the 0.24 mj/mm^2^-energy-applied group compared to the 0.12 mj/mm^2^-and 0.02 mj/mm^2^-applied groups [36]. 

In a very recent study, r-ESWT (*n* = 21 patients) and f-ESWT (*n* = 21 patients) types were compared for the first time in the literature in the treatment of knee OA. Although the shock and frequency values were the same in both groups, it was stated that f-ESWT was applied with 0.10 mj/mm^2^ energy and r-ESWT was applied with 3.0 bar pressure. Pain and physical function status assessed by VAS, WOMAC, and the 6 minute walk test in the 4th and 8th week of post-treatment controls improved significantly more in the f-ESWT group compared to the r-ESWT [21].

Except for knee joint dominance in the OA-ESWT studies, only one publication has addressed the treatment of carpometacarpal joint OA with ESWT. In this study, ESWT and intra-articular hyaluronic acid injection were compared, and it was revealed that they were similar in terms of VAS and Duruöz Hand Index scores, but superior treatment results were obtained in the ESWT group in the pinch test [37]. Success of shock wave treatment applied with a lower energy level in the carpometacarpal joint, which is a small joint of the upper extremity, may not be an appropriate criterion for hip OA. However, treatment of knee OA, which is a large joint of the lower extremity similar to the hip joint, with ESWT may constitute a more accurate model for our study. In the studies on the treatment of knee OA with ESWT [21,30,31,32,33,34,35,36], more positive treatment responses were obtained with higher energy and pressure values. However, the high level of energy might have affected the treatment results more positively than the high level of pressure [21]. The reality that f-ESWT was found to be more effective in our study may be related to the fact that the energy level reached 0.60 mj/mm^2^ and the pressure value reached 1.8 bar in certain sequences of the session. In our r-ESWT application, the energy level could only reach 0.192 mj/mm^2^, and the pressure value could reach up to 1.6 bars. Since the frequency level is up to 8 Hz and the number of shocks per session was 2000 in both our r-ESWT and f-ESWT applications, it can be accepted that these two factors did not play a role in the difference between the treatment results. In addition, f-ESWT, which can affect deeper tissues compared to r-ESWT [14], might have produced more effective results in the hip, which is a deep joint.

This study had some limitations. The treatment groups were non-homogeneous as they included both men and women. Since there had been no previous study of the treatment of hip OA with ESWT, the sample size was created by a calculation with software. Only X-ray radiographs (pelvic anterior posterior) were taken. Advanced imaging techniques such as USG, CT, or MRI were not used. In subsequent studies, advanced imaging methods can detect hip diseases such as bursitis, calcifications, ligament injuries, and so on, which may accompany hip OA. Only short-term outcomes were presented in this study. Similar studies can be conducted with longer follow-up periods.

## 5. Conclusions

In this trial, which was the first study of the treatment of human hip OA with ESWT, both the r-ESWT and f-ESWT were found to be more effective than the s-ESWT based on short-term outcomes. However, f-ESWT produced a superior improvement in follow-up parameters compared to r-ESWT.

## Figures and Tables

**Table 1:** Statistical analysis for participants’ characteristics.

	Focused ESWT (*n* = 51)	Radial ESWT (*n* = 49)	Sham ESWT (*n* = 48)	P1
Age (years)	63.7 ± 5.9 (51.5–75.0)	64.2 ± 6.8 (53.2–75.5)	63.4 ± 6.0 (54.1–75.3)	0.832
Gender (F/M)	33/18	33/16	32/16	0.959
BMI (kg/m^2^)	27.0 ± 1.7 (24.0–31.6)	26.8 ± 1.9 (23.0–31.5)	26.4 ± 2.7 (22.8–37.3)	0.389
Grade (II/III)	22/29	26/23	28/20	0.306
Pain duration (years)	2.8 ± 1.6 (0.5–8.2)	2.9 ± 1.7 (0.5–8.5)	2.7 ± 1.5 (0.6–7.8)	0.913

P1: Inter-group comparisons; ESWT: extracorporeal shock wave therapy; F/M: female/male; BMI: body mass index. Values are given as mean ± SD (min.–max.) or number. Grade: Kellgren–Lawrence classification.

**Table 2:** Statistical analysis for the VAS scores.

VAS	Focused ESWT (*n* = 51)	Radial ESWT (*n* = 49)	Sham ESWT (*n* = 48)	P1
(baseline)	7.4 ± 1.9 (4–10)	7.3 ± 1.9 (4–10)	7.2 ± 1.9 (4–10)	0.933
(4th week )	5.3 ± 2.1 (1–9)	6.3 ± 1.8 (3–10)	7.3 ± 2.1 (2–10)	
(8th week)	3.8 ± 1.9 (0–8)	5.5 ± 1.8 (2–10)	7.5 ± 1.9 (3–10)	
P2 (baseline vs. 4th week)	<0.001	<0.001	0.828	
P2 (baseline vs. 8th week)	<0.001	<0.001	0.070	
Change (baseline–4th week)	2.0 ± 1.7 (−1–8)	1.0 ± 1.1 (−2–3)	−0.0 ± 1.3 (−2–5)	0.002 ^‡^ <0.001 ^†,§^
Change (baseline—8th week)	3.6 ± 1.7 (0–8)	1.8 ± 1.3 (−2–5)	−0.3 ± 1.1 (−3–2)	<0.001 ^‡,†,§^

P1: Inter-group comparisons; P2: Intra-group comparisons; ^‡^: Focused vs. Radial; ^†^: Focused vs. Sham; ^§^: Radial vs. Sham. Data are expressed as mean ± SD; VAS: Visual Analog Scale.

**Table 3:** Statistical analysis for the WOMAC pain scores.

WOMAC Pain	Focused ESWT (*n* = 51)	Radial ESWT (*n* = 49)	Sham ESWT (*n* = 48)	P1
(baseline)	14.8 ± 4.1 (7–20)	14.6 ± 3.8 (7–20)	14.4 ± 4.0 (7–20)	0.877
(4th week)	10.5 ± 4.0 (3–17)	12.7 ± 3.3 (6–20)	14.3 ± 4.3 (4–20)	
(8th week)	7.5 ± 3.7 (1–15)	11.4 ± 3.8 (3–20)	15.0 ± 3.8 (7–20)	
P2 (baseline vs. 4th week)	<0.001	<0.001	0.862	
P2 (baseline vs. 8th week)	<0.001	<0.001	0.021	
Change (baseline–4th week)	4.3 ± 3.3 (0–14)	2.0 ± 1.7 (−2–6)	0.1 ± 2.5 (−3–10)	<0.001 ^‡,†,§^
Change (baseline–8th week)	7.3 ± 3.5 (0–16)	3.2 ± 2.5 (−3–10)	−0.6 ± 1.8 (−5–4)	<0.001 ^‡,†,§^

P1: Inter-group comparisons; P2: Intra-group comparisons; ^‡^: Focused vs. Radial; ^†^: Focused vs. Sham; ^§^: Radial vs. Sham. Data are expressed as mean ± SD; WOMAC: Western Ontario and McMaster Universities Osteoarthritis Index.

**Table 4:** Statistical analysis for the WOMAC stiffness scores.

WOMAC Stiffness	Focused ESWT (*n* = 51)	Radial ESWT (*n* = 49)	Sham ESWT (*n* = 48)	P1
(baseline)	5.4 ± 1.8 (2–8)	5.6 ± 1.7 (2–8)	5.3 ± 2.0 (2–8)	0.754
(4th week)	3.9 ± 1.9 (1–8)	4.7 ± 1.6 (2–8)	5.4 ± 2.3 (1–9)	
(8th week)	2.5 ± 1.6 (1–15)	4.0 ± 1.7 (1–8)	5.6 ± 2.0 (1–8)	
P2 (baseline vs. 4th week)	<0.001	<0.001	0.513	
P2 (baseline vs. 8th week)	<0.001	<0.001	0.031	
Change (baseline–4th week)	1.5 ± 1.6 (−4–5)	0.9 ± 1.0 (−2–3)	−0.1 ± 1.3 (−2–4)	0.002 ^‡^ <0.001 ^†,§^
Change (baseline–8th week)	2.9 ± 1.4 (0–7)	1.6 ± 1.2 (−2–4)	−0.3 ± 1.0 (−3–2)	<0.001 ^‡,†,§^

P1: Inter-group comparisons; P2: Intra-group comparisons; ‡: Focused vs. Radial; †: Focused vs. Sham; §: Radial vs. Sham. Data are expressed as mean ± SD; WOMAC: Western Ontario and McMaster Universities Osteoarthritis Index.

**Table 5:** Statistical analysis for the WOMAC function scores.

WOMAC Function	Focused ESWT (*n* = 51)	Radial ESWT (*n* = 49)	Sham ESWT (*n* = 48)	P1
(baseline)	48.7 ± 14.8 (19–68)	48.4 ± 14.2 (23–68)	48.0 ± 14.2 (23–68)	0.972
(4th week)	36.0 ± 14.4 (12–66)	43.1 ± 12.1 (22–62)	46.5 ± 14.3 (14–66)	
(8th week)	24.2 ± 11.6 (4–52)	38.1 ± 12.4 (10–60)	48.2 ± 14.6 (18–68)	
P2 (baseline vs. 4th week)	<0.001	<0.001	0.190	
P2 (baseline vs. 8th week)	<0.001	<0.001	0.850	
Change (baseline–4th week)	12.7 ± 10.5 (−5–49)	5.2 ± 4.3 (−3–15)	1.5 ± 8.0 (−11–34)	<0.001 ^‡,†^ <0.077 ^§^
Change (baseline–8th week)	24.5 ± 11.4 (1–44)	10.2 ± 7.5 (−2–32)	−0.2 ± 6.1 (−13–21)	<0.001 ^‡,†,§^

P1: Inter-group comparisons; P2: Intra-group comparisons; ‡: Focused vs. Radial; †: Focused vs. Sham; §: Radial vs. Sham. Data are expressed as mean ± SD; WOMAC: Western Ontario and McMaster Universities Osteoarthritis Index.

**Table 6:** Statistical analysis for the WOMAC total scores.

WOMAC Total	Focused ESWT (*n* = 51)	Radial ESWT (*n* = 49)	Sham ESWT (*n* = 48)	P1
(baseline)	68.9 ± 20.3 (31–96)	68.7 ± 19.3 (34–96)	67.7 ± 20.0 (33–96)	0.949
(4th week)	50.5 ± 19.2 (18–84)	60.5 ± 16.4 (33–85)	66.5 ± 20.1 (19–94)	
(8th week)	24.2 ± 11.6 (4–52)	53.8 ± 17.8 (16–86)	68.8 ± 20.3 (27–96)	
P2 (baseline vs. 4th week)	<0.001	<0.001	0.448	
P2 (baseline vs. 8th week)	<0.001	<0.001	0.375	
Change (baseline–4th week)	18.5 ± 14.5 (−6–68)	8.2 ± 5.9 (−5–20)	1.3 ± 11.3 (−14–47)	<0.001 ^‡,†,§^
Change (baseline–8th week)	35.2 ± 15.8 (2–65)	14.9 ± 10.8 (−5–45)	−1.1 ± 8.4 (−18–26)	<0.001 ^‡,†,§^

P1: Inter-group comparisons; P2: Intra-group comparisons; ‡: Focused vs. Radial; †: Focused vs. Sham; §: Radial vs. Sham. Data are expressed as mean ± SD; WOMAC: Western Ontario and McMaster Universities Osteoarthritis Index.

## Data Availability

The data presented in this study are available on request from the corresponding author. The data are not publicly available due to privacy issues.
